# Educational support and weekly telemonitoring during rapid maxillary expansion in children: a prospective preference-based comparative cohort study

**DOI:** 10.3389/fdmed.2026.1959672

**Published:** 2026-09-28

**Authors:** Sabina Saccomanno, Lorenzo Ederli Silenzi, Claudia Ciocia, Francesca Calò, Antonio Rizzo, Francesco Inchingolo, Jorida Jubani, Francesca Russo, Roberta Scarano, Grazia Marinelli, Alessio Danilo Inchingolo, Andrea Palermo, Marco Severino, Angelo Michele Inchingolo, Gianna Dipalma

**Affiliations:** 1 Department of Life Science Health and Health Profession, Link Campus University, Rome, Italy; 2 Department of Health Life and Environmental Science, University of L’Aquila, L’Aquila, Italy; 3 Interdisciplinary Department of Medicine, University of Bari “Aldo Moro”, Bari, Italy; 4 University of L'Aquila, L’Aquila, Italy; 5 Department of Innovative Technology in Medicine and Dentistry, University of Chieti-Pescara, Italy; 6 Department of Experimental Medicine, University of Salento, Lecce, Italy; 7 Department of Medicine and Surgery, University of Salento, Lecce, Italy

**Keywords:** caregiver, patient education, pediatric orthodontics, rapid maxillary expansion, telemonitoring, treatment adherence

## Abstract

**Background:**

Rapid maxillary expansion requires substantial caregiver participation in screw activation, oral hygiene and early recognition of appliance-related problems. Structured education and remote photographic monitoring may support families during the active expansion phase.

**Methods:**

This single-center, prospective, preference-based comparative cohort study included 100 children aged 7–11 years requiring rapid maxillary expansion. After receiving standardized information, caregivers independently selected conventional weekly in-office follow-up or an educational telemonitoring pathway comprising illustrated materials, standardized weekly caregiver-taken intraoral photographs acquired at home, clinician-led remote review and feedback and an in-office examination at completion of active expansion or earlier when clinically indicated. Quota-based consecutive recruitment continued until 50 participants were included in each cohort. The primary outcome was the proportion of participants with at least one recorded biological or technical complication during active expansion. All participants underwent the same standardized final in-office clinical examination.

**Results:**

All participants completed follow-up. In the telemonitoring cohort, 188 of 200 scheduled photographic sets were submitted, of which 174 (92.6%) were adequate for remote assessment. At least one recorded complication occurred in 12 of 50 participants (24.0%) in the telemonitoring cohort and 23 of 50 (46.0%) in the conventional cohort [unadjusted risk ratio [RR], 0.52; 95% confidence interval [CI], 0.29–0.93; *p* = 0.021]. Recorded biological complications occurred in 16.0% and 36.0% of participants, respectively (RR, 0.44; 95% CI, 0.21–0.93; *p* = 0.023), and recorded technical complications in 12.0% and 30.0%, respectively (RR, 0.40; 95% CI, 0.17–0.95; *p* = 0.027). Mean activation adherence was higher in the telemonitoring cohort (94.8 ± 6.1% vs. 89.3 ± 9.7%; mean difference, 5.5 percentage points; 95% CI, 2.3–8.7; *p* = 0.001). Child- and caregiver-reported treatment acceptance scores were also higher. Unscheduled in-office visits did not differ significantly between cohorts, and no delayed detection of clinically relevant complications occurred

**Conclusions:**

The educational telemonitoring pathway was feasible and associated with fewer recorded complications, higher activation adherence, and greater treatment acceptance. However, because caregivers selected the follow-up pathway, estimates were unadjusted, and interim assessment modalities differed between cohorts, the findings should be considered exploratory and not interpreted as evidence of a causal treatment effect.

## 1 Introduction

Rapid maxillary expansion is a widely used orthopedic procedure in growing patients for the correction of transverse maxillary deficiency, posterior crossbite, dental crowding, and selected functional conditions associated with reduced nasal airflow or altered oral function. Its skeletal and dentoalveolar effects have been extensively documented, particularly when treatment is performed during childhood and early adolescence, when the midpalatal suture remains responsive to orthopedic forces (1–7). Although rapid maxillary expansion is clinically effective, its active phase requires substantial caregiver involvement. Parents or legal guardians are commonly responsible for screw activation, supervision of oral hygiene and early recognition of appliance-related or soft tissue problems. Missed or incorrect activations, uncertainty regarding screw progression, plaque accumulation, mucosal irritation, persistent discomfort or appliance loosening may interfere with treatment progression and may require additional clinical assessment (8–14). Conventional management generally includes verbal instructions at appliance delivery and regular in-office follow-up. However, verbal information may be incompletely retained, particularly when caregivers are required to perform repeated technical procedures at home (15–20). Written and visual educational support may improve caregiver understanding and confidence, while remote monitoring may facilitate communication with the orthodontic team and the early identification of possible problems between scheduled appointments (21–27). Teleorthodontics has gained increasing attention as an adjunct to conventional clinical care. Standardized intraoral photographs taken by patients or caregivers may provide clinically useful information regarding oral hygiene, soft tissue conditions, appliance integrity, and treatment progression. During rapid maxillary expansion, remote photographic assessment may be particularly applicable because the expansion screw, palatal tissues, midline diastema and visible aspects of appliance stability can often be evaluated through standardized intraoral images (28–38). Nevertheless, photographic assessment cannot reproduce palpation, appliance mobility testing or a complete chairside examination and should therefore be regarded as a supportive monitoring and triage tool rather than a replacement for clinical evaluation.

Despite the growing interest in digital orthodontic monitoring, evidence regarding simple, non-commercial, clinician-led telemonitoring protocols during rapid maxillary expansion remains limited. Many available systems rely on dedicated platforms or artificial intelligence-supported software that may not be accessible in all clinical settings. A pragmatic pathway based on structured caregiver education, standardized home photography secure image transmission and clinician-led remote review may represent an accessible approach for selected families undergoing pediatric orthodontic treatment (39–49).

Therefore, the primary objective of this study was to compare the frequency of recorded biological and technical complications during active rapid maxillary expansion between two caregiver-selected follow-up pathways: conventional weekly in-office follow-up and an educational telemonitoring pathway. Secondary objectives were to compare activation adherence, child- and caregiver-reported treatment acceptance, adequacy of remote photographic assessment, unscheduled in-office visits, treatment interruption and delayed detection of clinically relevant complications (50–57). We hypothesized that selection of the educational telemonitoring pathway would be associated with a fewer recorded biological and technical complications, higher activation adherence, greater treatment acceptance, without an increased frequency of unscheduled visits or delayed detection of clinically relevant complications. Because participants were not randomly assigned and the frequency and modality of interim assessment differed between the two pathways, all between-cohort comparisons were considered exploratory and were not intended to establish causal effectiveness (58–68).

## 2 Materials and methods

### 2.1 Study design

This was a single-center, prospective, observational, preference-based comparative cohort study involving children undergoing rapid maxillary expansion. The study compared two follow-up pathways available in routine clinical practice during the active expansion phase: conventional weekly in-office follow-up and an educational telemonitoring pathway based on structured caregiver education, standardized weekly intraoral photographs, clinician-led remote review and in-office reassessment when clinically indicated.

Participants were enrolled between November 2025 and March 2026 at the Orthodontics Clinic of the Policlinico di Bari, Bari, Italy. Potentially eligible children were identified among patients for whom rapid maxillary expansion had already been clinically indicated as part of routine orthodontic care. The indication for treatment and the selection of the follow-up pathway were established independently of study participation. During routine clinical counseling, all eligible families received the same standardized information regarding the characteristics, requirements and limitations of the two available follow-up pathways. Caregivers independently selected their preferred pathway before being invited to participate in the study. The investigators did not assign, influence or redirect families toward either pathway. Only after the follow-up pathway had been selected were families invited to participate in the observational study. Written informed consent was obtained before prospective study-related data collection began.

Participants were followed prospectively from appliance delivery until completion of the active expansion phase. The frequency and modality of interim assessment differed according to the selected pathway: participants in the conventional cohort underwent weekly chairside examinations, whereas participants in the educational telemonitoring cohort primarily underwent weekly photographic review and attended an in-office examination at completion of active expansion or earlier when clinically indicated. To improve comparability of the final assessment, all participants underwent the same standardized in-office clinical examination at completion of active expansion. Nevertheless, because transient events may have had different probabilities of detection and documentation during follow-up, all between-cohort comparisons of recorded complications were considered exploratory.

All clinical procedures and in-office assessments were performed at the same institution by orthodontists experienced in pediatric orthodontics and rapid maxillary expansion. Reporting followed the Strengthening the Reporting of Observational Studies in Epidemiology (STROBE) recommendations, while the educational telemonitoring pathway was described according to the Template for Intervention Description and Replication (TIDieR) principles. Patients, caregivers and members of the public were not involved as research partners in formulating the research question, selecting outcomes, analyzing the data or preparing the manuscript. Their involvement was limited to selecting one of the clinically available follow-up pathways, participating in the study and completing the required procedures and assessments.

### 2.2 Inclusion criteria

Patients were eligible for inclusion if they met all of the following criteria:

1. Age between 7 and 11 years at the time of appliance delivery;
2. Mixed dentition;
3. Clinical indication for rapid maxillary expansion because of transverse maxillary deficiency, with or without posterior crossbite;
4. No previous treatment with a maxillary expansion appliance;
5. Availability of at least one parent or legal guardian able to understand the activation protocol and comply with the requirements of the independently selected follow-up pathway;
6. Willingness and ability of caregivers to perform home screw activation according to the prescribed protocol and to complete the daily activation log;
7. For families selecting the educational telemonitoring pathway, availability of a smartphone suitable for standardized intraoral photography and access to the approved secure communication channel for image transmission;
8. Willingness of caregivers in the telemonitoring pathway to acquire and submit standardized weekly intraoral photographs according to the study protocol;
9. Written informed consent provided by parents or legal guardians before enrollment and before the beginning of prospective study-related data collection;
10. Child assent obtained when appropriate, according to age and level of understanding.

### 2.3 Exclusion criteria

Patients were excluded if one or more of the following conditions were present:

1. Craniofacial syndromes, cleft lip and/or palate or other congenital craniofacial anomalies;
2. Systemic diseases or current medications potentially affecting oral tissues, bone metabolism, wound healing or orthodontic treatment response;
3. Severe periodontal or mucogingival conditions contraindicating appliance placement;
4. Poor oral hygiene at baseline requiring preliminary improvement before rapid maxillary expansion;
5. Need for simultaneous orthodontic or orthopedic procedures during the active expansion phase that could interfere with assessment of the study outcomes;
6. Inability of the caregivers to understand or perform the prescribed screw activation protocol;
7. Inability of the child or caregiver to comply with the requirements of the independently selected follow-up pathway;
8. Refusal or inability of the parent or legal guardian to provide written informed consent;
9. For families selecting the educational telemonitoring pathway, inability or unwillingness to acquire and submit standardized intraoral photographs through the approved secure communication channel.

### 2.4 Selection of the follow-Up pathway and cohort formation

Participants were not randomly assigned to the study cohorts. After the indication for rapid maxillary expansion had been confirmed, all eligible families received the same standardized explanation of the two follow-up pathways available in routine clinical practice. Detailed procedures for the two follow-up pathways are described in Section 2.6.

Caregivers independently selected the pathway they considered most appropriate for their family. The investigators did not assign, influence or redirect participants to either pathway. The selected follow-up pathway was recorded at study enrollment, after the family had made its clinical choice and before prospective study-related data collection began. Participants were subsequently followed and analyzed within the corresponding preference-based cohort.

Quota-based consecutive recruitment was conducted separately within each caregiver-selected pathway. The first 50 eligible and consenting participants who selected conventional weekly in-office follow-up were included in the conventional cohort, whereas the first 50 eligible and consenting participants who selected the educational telemonitoring pathway were included in the telemonitoring cohort. Recruitment within each cohort was closed once the prespecified target of 50 participants had been reached. Families selecting a pathway after the corresponding recruitment quota had been reached continued to receive their preferred clinical follow-up but were not enrolled in the study.

Therefore, the enrolled population represented two consecutive samples recruited within the respective caregiver-selected pathways rather than a single consecutive clinical sample.

Because of the nature of the follow-up pathways, participants, caregivers and treating clinicians could not be blinded. At completion of active expansion, all participants underwent the same standardized in-office clinical examination. The findings recorded during this final assessment were independently reviewed by two examiners who were not directly involved in routine treatment delivery. However, complete masking to cohort allocation could not be guaranteed because the clinical records and photographic documentation differed between pathways. Any disagreement regarding the identification or classification of a clinical finding was resolved through discussion and consensus. No formal inter-examiner reliability analysis was performed. The different frequency and modality of interim assessment were inherent characteristics of the two clinical pathways. Consequently, minor or transient findings may have had different probabilities of detection and documentation between cohorts. For this reason, comparisons of recorded complications were considered exploratory and were interpreted together with the results of the standardized final in-office examination.

### 2.5 Study size

No formal *a priori* sample size calculation was performed. Before recruitment began, a pragmatic target of 50 participants per cohort was established, resulting in a planned total sample of 100 participants. This target was selected on the basis of the number of potentially eligible patients expected during the predefined recruitment period and the feasibility of completing prospective follow-up within a single clinical center.

To characterize the statistical sensitivity of the achieved sample, a sensitivity analysis was subsequently performed for the binary primary outcome. Assuming equal group sizes, a two-sided alpha level of 0.05% and 80% statistical power, 50 participants per cohort correspond to a minimum detectable standardized difference in proportions of approximately Cohen's *h* = 0.56. Using an event proportion of approximately 46% as a reference, this corresponds to a detectable event proportion of approximately 20% in the comparison cohort, or an absolute between-cohort difference of approximately 26 percentage points. Thus, the achieved sample size was primarily capable of detecting relatively large between-cohort differences and may have had insufficient statistical power for smaller effects or uncommon outcomes.

Because the study size was not based on *a priori* effect-size assumption, all comparative analyses were considered exploratory. Effect estimates were therefore reported with 95%confidence intervals and the absence of a statistically significant difference was not interpreted asevidence of equivalence or absence of an association.

### 2.6 Follow-Up pathways

#### 2.6.1 Conventional follow-Up cohort

Participants in the conventional follow-up cohort received standardized verbal instructions and a chairside demonstration regarding the prescribed screw-activation schedule, oral-hygiene procedures, expected discomfort, dietary precautions, and warning signs requiring prompt contact with the orthodontic team.

Participants attended scheduled weekly in-office assessments throughout the active expansion phase. At each appointment, the orthodontist evaluated appliance integrity and stability, screw-activation progression, oral hygiene, palatal and gingival soft tissue conditions, caregiver-reported symptoms and achievement of the planned transverse correction. Additional clinical management or an unscheduled assessment was provided whenever a biological or technical complication was identified or suspected.

Participants in this cohort did not receive the illustrated educational booklet, photographic mouth opener, written home-photography protocol, routine weekly image submission or structured remote photographic review provided to the educational telemonitoring cohort. Communication with the orthodontic team between scheduled visits remained available whenever caregivers reported pain, difficulty activating the screw, appliance loosening or other concerns.

At completion of active expansion, all participants underwent the same standardized final in-office clinical examination used for the educational telemonitoring cohort.

#### 2.6.2 Educational telemonitoring cohort

Participants in the educational telemonitoring cohort received the same standardized verbal instructions and chairside demonstration provided to participants in the conventional follow-up cohort. Caregivers were instructed regarding the prescribed screw-activation schedule, oral-hygiene procedures, expected discomfort, dietary precautions and warning signs requiring prompt contact with the orthodontic team. In addition, they received a structured educational package consisting of an illustrated instruction booklet, a photographic mouth opener and a written protocol for standardized home intraoral photography (Supplementary Figures A1, A2, Appendix A). The educational materials described the activation procedure, oral-hygiene recommendations, photographic technique, image-quality requirements and clinical warning signs requiring professional assessment.

Caregivers were asked to acquire and submit one set of standardized intraoral photographs each week during the active expansion phase. The submitted images were reviewed by the orthodontist to assess visible oral hygiene conditions, palatal and gingival soft tissue, appliance integrity, screw visibility and activation progression and possible warning signs. Caregivers received remote feedback when clarification, reinforcement of the activation protocol, improvement in oral hygiene, or repeat photographs were required.

Participants underwent a scheduled in-office examination at completion of active expansion. When active expansion extended beyond four weeks, an additional in-office reassessment was scheduled at approximately four weeks. Earlier unscheduled visits were arranged whenever the photographic assessment was inconclusive, image quality was inadequate or a possible biological or technical complication was identified or suspected.

### 2.7 Rapid maxillary expansion protocol

All participants were treated with a fixed rapid maxillary expander. A Hyrax- or Haas-type appliance was selected according to the individual clinical indication and was anchored on the first permanent molars and/or second deciduous molars, depending on dental development and the availability of suitable anchorage teeth. Appliance selection was performed independently of the chosen follow-up pathway.

The screw-activation protocol was identical in both cohorts. At appliance delivery, caregivers were instructed to activate the screw by one quarter-turn twice daily during the first week, followed by one quarter-turn once daily until the planned transverse correction was achieved. Caregivers in both cohorts were required to record each prescribed activation in the daily activation log.

Active expansion was continued until correction of the transverse discrepancy, defined clinically as correction of posterior crossbite and/or achievement of slight transverse overcorrection, with the palatal cusps of the maxillary posterior teeth contacting or slightly overlapping the buccal cusps of the mandibular posterior teeth. The decision to discontinue active screw activation was based on the clinical assessment of the treating orthodontist.

At completion of active expansion, the expansion screw was blocked and the appliance was maintained in place as a passive retainer for 6 months. The outcomes evaluated in the present study referred exclusively to the active expansion phase, from appliance delivery to completion of active screw activation. No additional orthodontic or orthopedic procedures that could interfere with assessment of the study outcomes were performed during this period.

### 2.8 Telemonitoring workflow

In the educational telemonitoring cohort, four standardized photographic sets were scheduled for each participant during the active expansion phase. The first set was acquired at appliance delivery and the subsequent sets were scheduled approximately 7, 14, and 21 days later. This protocol resulted in 200 expected photographic sets across the 50 participants. When active expansion was completed before the last scheduled submission, the final photographic set was acquired immediately before or at completion of active expansion. Additional photographs could be requested whenever image quality was inadequate or clarification of a possible clinical finding was required.

Photographs were acquired at home by the caregiver using a smartphone camera and the photographic mouth opener provided at appliance delivery. Each photographic set included the following four intraoral views:

1. A frontal intraoral view in light occlusion;
2. An upper occlusal view showing the expander and screw;
3. A right lateral intraoral view;
4. A left lateral intraoral view.

A photographic set was considered adequate for remote assessment when the submitted images were sufficiently clear and complete to permit visual evaluation of screw visibility and activation progression, visible appliance integrity, oral-hygiene conditions, and palatal and gingival soft tissues without immediate chairside verification. The upper occlusal view was considered essential for assessing the expansion screw, palatal tissues, visible plaque accumulation, and possible changes in appliance position. Photographic assessment was limited to findings that could be evaluated visually and did not permit palpation, appliance mobility testing or a complete clinical examination.

Caregivers were instructed to acquire the photographs after toothbrushing, preferably on the same day each week, using adequate lighting and avoiding blurred, incomplete or poorly centered images. The illustrated acquisition instructions and image-quality criteria are provided in (Supplementary Figure A2) and Appendix A. When one or more images were inadequate, the caregiver was contacted and asked to repeat the relevant photograph.

Images were submitted through an approved secure clinical messaging system or encrypted institutional e-mail, according to institutional privacy and data protection regulations. Files were pseudonymized using the participant identification code and submission date. No facial photographs were required.

Each photographic set was reviewed by the orthodontist within 24–48 h of submission. The review focused on visible oral-hygiene conditions, gingival or palatal redness, swelling or ulceration, screw visibility and activation progression, possible appliance deformation or loosening and any other warning signs reported by the caregiver. For each review, image adequacy, visible findings, caregiver contact, requests for repeat photographs, remote instructions and the decision to arrange an in-office reassessment were documented.

Caregivers were contacted when the images showed or suggested mucosal ulceration, swelling, marked redness, clinically relevant plaque accumulation, uncertain or incorrect screw activation, possible appliance loosening or deformation or insufficient image quality. In such cases, additional photographs were requested and/or an unscheduled in-office examination was arranged when chairside verification was considered necessary. Findings suspected through photographic review were therefore treated as screening or triage findings rather than definitive clinical diagnoses.

Telemonitoring was used as a supportive monitoring and triage strategy and did not replace clinical examination when a biological or technical complication was suspected. Families were instructed to contact the orthodontic team promptly in the event of persistent pain, bleeding, appliance detachment, inability to activate the screw, sudden changes in appliance position or any other urgent concern. At completion of active expansion, all participants underwent the same standardized final in-office clinical examination performed in the conventional follow-up cohort.

### 2.9 Outcome measures

#### 2.9.1 Primary outcome

The primary outcome was the proportion of participants experiencing at least one recorded biological or technical complication during the active rapid maxillary expansion phase. A recorded complication was defined as an event meeting the prespecified criteria described below and documented prospectively through scheduled clinical assessments, caregiver communications, remote photographic review, unscheduled in-office assessments or the standardized final in-office examination.

Biological complications included gingival inflammation, palatal or buccal mucosal irritation or ulceration, soft tissue swelling, pain persisting for more than 3 days and clinically relevant plaque accumulation around the appliance requiring additional reinforcement of oral hygiene instructions or clinical intervention.

Technical complications included expander loosening, band or cement failure, incorrect or uncertain screw activation, inability to activate the screw, suspected appliance deformation or instability and the need for appliance repair or recementation.

The primary outcome was analyzed at the participant level. Each participant was classified as having or not having at least one recorded complication during active expansion. Participants experiencing multiple events were counted only once in the composite primary outcome, while biological and technical complications were also recorded and analyzed separately. Because a participant could experience both a biological and a technical complication, the sum of the individual complication categories could exceed the number of participants included in the composite outcome.

All participants underwent the same standardized final in-office clinical examination at completion of active expansion. However, because the frequency and modality of interim assessment differed between the two follow-up pathways, minor or transient complications may have had different probabilities of detection and documentation. The primary outcome therefore represented the frequency of recorded complications rather than a definitive estimate of the true incidence of all complications and was interpreted as an exploratory comparative outcome.

#### 2.9.2 Secondary outcomes

Secondary outcomes included recorded biological complications, recorded technical complications, activation-related problems, activation adherence, child-reported treatment acceptance, caregiver- reported treatment acceptance, caregiver confidence in appliance management, overall treatment satisfaction, adequacy of remote photographic assessment, unscheduled in-office visits, temporary treatment interruption, treatment discontinuation and delayed detection of clinically relevant complications. All secondary between-cohort comparisons were considered exploratory.

Activation adherence was calculated for each participant as the number of prescribed screw activations documented as correctly completed in the caregiver activation log divided by the total number of prescribed activations during the active expansion phase, multiplied by 100. Caregivers in both cohorts were instructed to record each activation in a daily activation log. The logs were reviewed during scheduled or final in-office assessments and compared with the clinically observed progression of the expansion screw. Missed or incorrectly performed activations were classified as non-adherent.

In the educational telemonitoring cohort, serial upper occlusal photographs provided an additional supportive source for assessing screw visibility and activation progression. However, photographic verification was not available in the conventional cohort and did not replace the standardized adherence calculation based on caregiver activation logs and clinical screw progression. Consequently, differential assessment of activation adherence between cohorts could not be completely excluded.

Treatment acceptance and satisfaction-related outcomes were assessed at completion of active expansion using study-specific patient- and caregiver-reported items developed for the purposes of the present study rather than a previously validated psychometric instrument. Each construct was assessed separately using a single item scored on a 5-point Likert scale, with higher scores indicating greater acceptance, confidence, or satisfaction. The child assessment included one item evaluating overall treatment acceptance. The caregiver assessment included separate single items evaluating treatment acceptance, confidence in appliance management, and overall treatment satisfaction. No formal psychometric validation, construct-validation study, or test–retest reliability assessment was performed. Because the individual constructs were each assessed using a single item rather than a multi-item scale, internal-consistency coefficients such as Cronbach's alpha were not applicable. These patient- and caregiver-reported outcomes were therefore considered exploratory.

Adequacy of remote photographic assessment was evaluated only in the educational telemonitoring cohort. It was calculated at the photographic-set level as the proportion of submitted weekly sets considered sufficiently clear and complete to permit visual assessment of screw visibility and activation progression, visible appliance integrity, oral-hygiene conditions, and palatal and gingival soft tissues without immediate chairside verification. Photographic submission rate was calculated using the total number of expected photographic sets as the denominator, whereas photographic adequacy was calculated using the number of submitted sets as the denominator.

An unscheduled in-office visit was defined as any additional chairside assessment prompted by pain, caregiver concern, suspected biological or technical complications, possible appliance instability, uncertain screw activation, inconclusive remote findings or inadequate image quality. Because the reasons leading to an unscheduled visit could differ according to the selected follow-up pathway, this outcome was interpreted as a measure of additional chairside assessment rather than as a direct measure of complication frequency.

Temporary treatment interruption and treatment discontinuation during the active expansion phase were documented from the clinical records. Delayed detection was defined as a clinically relevant biological or technical complication that was not identified during the applicable scheduled or remote follow-up and was subsequently judged to have required earlier clinical intervention. Assessment of delayed detection was based on review of the prospective follow-up documentation, caregiver communications, clinical records and the standardized final in-office examination.

### 2.10 Data collection

Baseline data were collected before the selected follow-up pathway began and included age, sex, dentition stage, clinical indication for rapid maxillary expansion, presence or absence of posterior crossbite, type of expansion appliance and the follow-up pathway independently selected by the caregiver.

Follow-up data were collected prospectively from appliance delivery until completion of the active expansion phase. Caregiver activation logs were collected and reviewed to determine the number of prescribed screw activations documented as correctly completed for each participant. Log entries were compared with the clinically observed progression of the expansion screw during scheduled, unscheduled or final in-office assessments.

Clinical records were used to document screw progression, recorded biological and technical complications, activation-related problems, caregiver-reported concerns, scheduled and unscheduled assessments, temporary treatment interruption, treatment discontinuation and completion of active expansion. Complication ascertainment could draw on the sources available within each follow-up pathway, including scheduled clinical examinations, caregiver communications, remote photographic review, unscheduled in-office assessments and the standardized final clinical examination. However, the study database did not prospectively encode a mutually exclusive source-of-detection variable assigning each participant-level complication to a single detection source. Because the same event could be reported or identified through more than one source and subsequently confirmed clinically, retrospective attribution of individual complications exclusively to interim assessment or to the final examination was not considered sufficiently reliable for formal stratified analysis.

Pathway-specific follow-up data were collected according to the procedures described in Sections 2.6 and 2.8. For the conventional follow-up cohort, data from scheduled in-office assessments were recorded in the clinical records. For the educational telemonitoring cohort, additional telemonitoring-specific data included photographic submission dates, image adequacy, visible clinical findings, requests for repeat photographs, caregiver contact, remote instructions, and decisions to arrange an unscheduled in-office assessment.

At completion of active expansion, all participants underwent the same standardized final in-office clinical examination. This assessment included evaluation of oral hygiene, gingival and palatal soft tissues, screw position and activation progression, appliance integrity and stability and the presence of biological or technical complications. Child- and caregiver-reported questionnaires were also collected at completion of active expansion.

Data were entered into a dedicated pseudonymized database by a trained member of the research team and were independently checked for completeness and accuracy by a second member of the research team. Discrepancies were resolved by reviewing the original clinical records, caregiver activation logs, questionnaires or photographic documentation, as applicable. Access to the database was restricted to authorized research-team members.

Because interim data were obtained through different assessment modalities in the two cohorts, the collected data represented prospectively documented or recorded events rather than a definitive measure of the true incidence of all transient complications. The standardized final in-office examination improved comparability between cohorts but could not identify minor events that had resolved before the final assessment.

### 2.11 Statistical analysis

Descriptive statistics were calculated for study variables. Continuous variables were summarized as mean and standard deviation or as median and interquartile range, according to their distribution. Categorical variables were reported as absolute frequencies and percentages. The distribution of continuous variables was assessed using the Shapiro–Wilk test and visual inspection of the corresponding distributions.

Because allocation to the follow-up pathways was based on caregiver preference rather than randomization, measured baseline characteristics were compared descriptively using standardized mean differences (SMDs). SMDs were calculated as the value in the educational telemonitoring cohort minus the corresponding value in the conventional follow-up cohort. Absolute SMD values of 0.10 or greater were considered indicative of a potentially meaningful between-cohort difference. No significance tests were performed to assess baseline equivalence and small SMDs were not interpreted as evidence that the cohorts were fully comparable with respect to measured or unmeasured characteristics.

All participants were analyzed within the follow-up pathway independently selected by their caregivers. Categorical outcomes were compared using Pearson's chi-square test. Fisher's exact test was used when one or more expected cell counts were lower than 5. Continuous outcomes were compared using independent-samples t-tests when the assumptions for parametric analysis were satisfied; otherwise, the Mann–Whitney U test was used.

For binary outcomes, unadjusted risk ratios (RRs) and corresponding 95% confidence intervals (CIs) were calculated using the conventional follow-up cohort as the reference group. An RR below 1 therefore indicated a lower frequency of the recorded outcome in the educational telemonitoring cohort. For continuous outcomes, unadjusted mean differences and corresponding 95% CIs were calculated as the mean value in the educational telemonitoring cohort minus the mean value in the conventional follow-up cohort.

The primary analysis concerned the participant-level composite outcome of at least one recorded biological or technical complication during active expansion. Separate analyses of recorded biological complication, recorded technical complications, activation-related problems, activation adherence, treatment-acceptance measures, unscheduled in-office visits and treatment interruptions were considered secondary and exploratory. A source-stratified sensitivity analysis comparing complications detected during pathway-specific interim assessments with those detected at the standardized final examination was not performed because a mutually exclusive source-of-detection variable had not been prospectively encoded for each participant-level complication. Retrospective reconstruction from overlapping clinical documentation was considered potentially prone to misclassification.

No multivariable adjusted analysis was performed. For the primary binary outcome, only 35 participants experienced the composite event, limiting the number of covariates that could be included without materially increasing the risk of unstable estimates and model overfitting. In addition, several important potential confounders related to caregiver pathway selection and patient- or caregiver-reported outcomes, including caregiver motivation, digital competence, health literacy, socioeconomic circumstances, and treatment expectations, were not comprehensively measured. Consequently, an adjusted model restricted to the available demographic and clinical variables would not have adequately addressed these major sources of residual confounding. All comparative effect estimates were therefore reported unadjusted and were interpreted as exploratory observational associations rather than causal treatment effects.

Because the frequency and modality of interim assessment differed between cohorts, the comparison of recorded complications was potentially affected by differential outcome ascertainment. The standardized final in-office examination performed in all participants improved the comparability of the final assessment but could not identify minor or transient events that had resolved before treatment completion. Therefore, the statistical comparison of recorded complications was not interpreted as a definitive estimate of the true difference in complication incidence between the two follow-up pathways.

No adjustment for multiple comparisons was applied. Accordingly, *p*-values for secondary outcomes were considered exploratory and were interpreted cautiously. All statistical tests were two-sided and a *p*-value below 0.05 was considered statistically significant. Statistical significance was not interpreted as evidence of clinical importance or causality.

No imputation was performed because primary outcome data were available for all included participants. The photographic submission rate was calculated using the total number of expected photographic sets as the denominator, whereas photographic adequacy was calculated using the number of submitted photographic sets as the denominator. Analyses were performed using IBM SPSS Statistics, version 28.0 (IBM Corp., Armonk, NY, USA).

### 2.12 Ethical considerations

The study was conducted in accordance with the principles of the Declaration of Helsinki and was approved by the competent Ethics Committee (protocol code 2427/CEL; approval date: 1 October 2025).

Written informed consent was obtained from the parents or legal guardians of all participants before enrollment and before the beginning of prospective study-related data collection. Child assent was also obtained when appropriate, according to the child's age and level of understanding.

The clinical indication for rapid maxillary expansion and the caregiver's selection of the follow-up pathway were established as part of routine clinical decision-making and independently of study participation. Families selected their preferred pathway before being invited to participate in the observational study. Refusal to participate in the study did not affect the clinical care provided or the family's previously selected follow-up pathway.

For participants in the educational telemonitoring cohort, specific informed consent was obtained for the acquisition, transmission, storage and study-related use of intraoral photographs. Caregivers were informed about the purpose of photographic monitoring, the procedures for secure image transmission, the limits of remote assessment and the circumstances requiring an in-office clinical examination. They were also informed that telemonitoring was intended as a supportive monitoring and triage strategy and did not replace chairside evaluation when clinically indicated.

Clinical and photographic data were pseudonymized using a participant identification code. Images were transmitted through an approved secure clinical messaging system or encrypted institutional email in accordance with institutional privacy and data-protection procedures. No facial photographs were required and no directly identifiable patient images were included in the manuscript. Access to clinical, photographic and study data was restricted to authorized members of the research team.

## 3 Results

### 3.1 Study population and baseline characteristics

A total of 168 patients were assessed for eligibility. Eight patients were excluded before cohort recruitment, leaving 160 eligible families who independently selected one of the two follow-up pathways available in routine clinical practice. Among these, 84 families selected conventional weekly in-office follow-up and 76 selected the educational telemonitoring pathway.

Quota-based consecutive recruitment was conducted separately within each caregiver-selected pathway. The first 50 eligible and consenting participants who selected conventional follow-up were included in the conventional cohort, whereas the first 50 eligible and consenting participants who selected educational telemonitoring were included in the telemonitoring cohort. After the prespecified quota of 50 participants had been reached in each cohort, 34 additional families who had selected conventional follow-up and 26 additional families who had selected educational telemonitoring were not enrolled in the study but continued to receive their independently selected clinical follow-up pathway. No participant was assigned, redirected or encouraged to select a particular pathway by the investigators.

All 100 enrolled participants completed the active expansion phase and underwent the standardized final in-office clinical examination. Primary outcome data were available for all participants and no participant was excluded from the final analysis. Two participants in the conventional cohort experienced a temporary interruption of the activation protocol; both remained under follow-up and were included in the analysis. Participant flow is shown in Figure 1.

**Figure 1:**
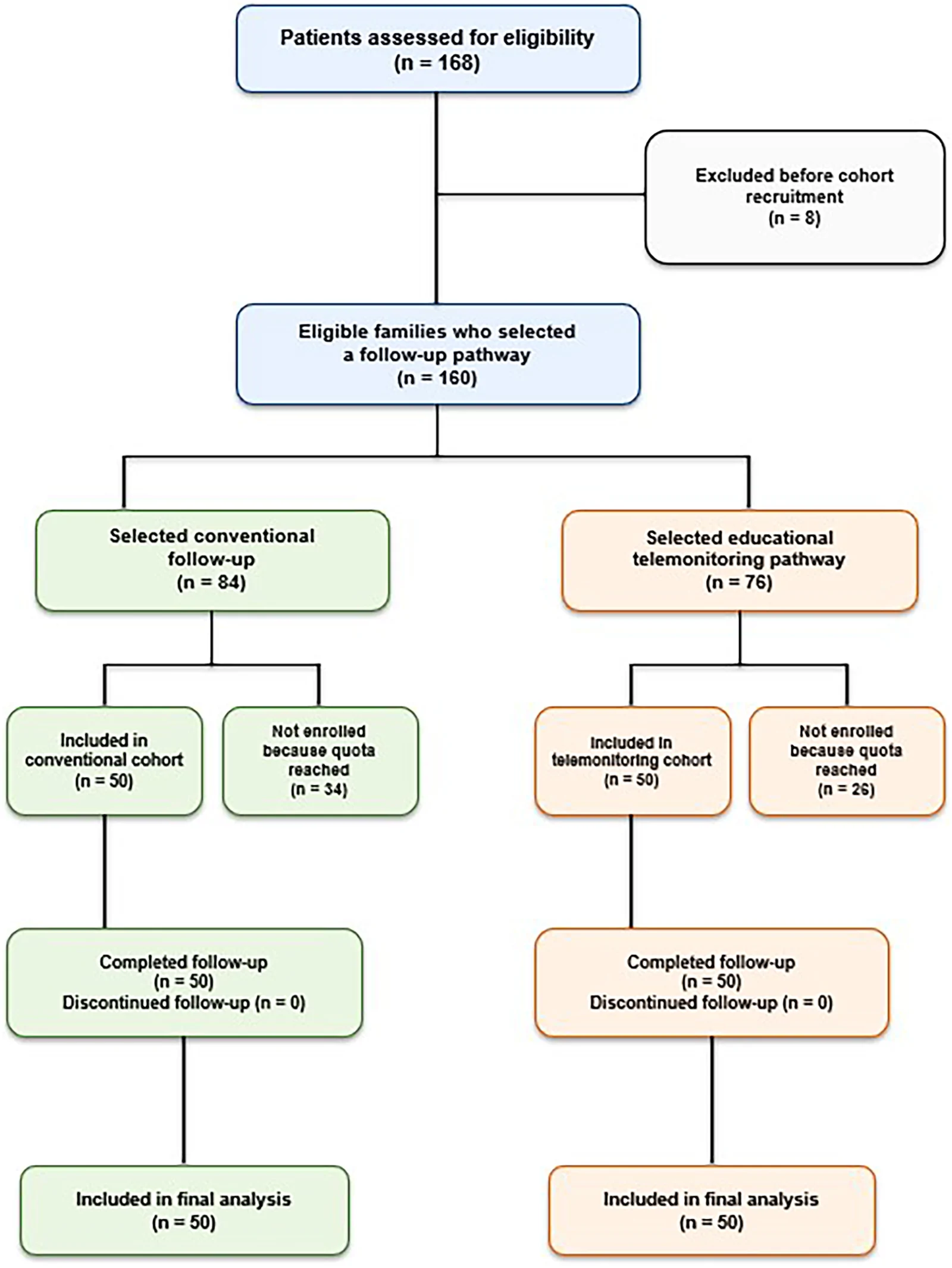
Participant flow through the prospective preference-based comparative cohort study.

Mean age was 8.6 ± 1.1 years in the conventional cohort and 8.7 ± 1.0 years in the educational telemonitoring cohort. The absolute standardized mean difference for age was 0.10, reaching the prespecified threshold for a potentially meaningful difference. The proportion of female participants was 44.0% in the conventional cohort and 48.0% in the telemonitoring cohort. Posterior crossbite was present in 78.0% and 76.0% of participants, respectively, while a Hyrax expander was used in 92.0% and 90.0%, respectively. All participants were in mixed dentition and followed the same screw-activation protocol.

The measured baseline demographic and clinical characteristics are presented in Table 1.

**Table 1:** Baseline demographic and clinical characteristics of the caregiver-selected follow-up cohorts.

Variable	Conventional cohort (*n* = 50)	Educational telemonitoring cohort (*n* = 50)	SMD
Age, years, mean ± SD	8.6 ± 1.1	8.7 ± 1.0	0.10
Female sex, *n* (%)	22 (44.0)	24 (48.0)	0.08
Posterior crossbite, *n* (%)	39 (78.0)	38 (76.0)	−0.05
Hyrax expander, *n* (%)	46 (92.0)	45 (90.0)	−0.07

Data are presented as mean ± standard deviation or number and percentage. Standardized mean differences were calculated as the value in the educational telemonitoring cohort minus the corresponding value in the conventional follow-up cohort. An absolute SMD of 0.10 or greater was considered indicative of a potentially meaningful between-cohort difference. SMDs were used descriptively and were not interpreted as statistical tests of baseline equivalence. SD, standard deviation; SMD, standardized mean difference.

### 3.2 Treatment duration and feasibility of weekly telemonitoring

The mean duration of the active expansion phase was 22.8 ± 4.3 days in the conventional follow-up cohort and 22.1 ± 4.1 days in the educational telemonitoring cohort. The unadjusted mean difference, calculated as the educational telemonitoring cohort minus the conventional cohort, was −0.7 days (95% CI, −2.37 to 0.97; *p* = 0.407). Thus, no statistically significant difference in active expansion duration was observed between the two cohorts.

Four standardized photographic sets were scheduled for each of the 50 participants in the educational telemonitoring cohort, resulting in a total of 200 expected photographic sets. The sets were scheduled at appliance delivery and approximately 7, 14, and 21 days thereafter. When active expansion was completed before the last planned submission, the final photographic set was acquired immediately before or at completion of active expansion.

Caregivers submitted 188 of the 200 expected photographic sets, corresponding to a submission rate of 94.0%. Of the 188 submitted sets, 174 (92.6%) met the predefined adequacy criteria for remote visual assessment. Fourteen sets were considered inadequate, most commonly because of insufficient screw visibility, image blurring, incomplete intraoral views, poor image centering, or inadequate lighting.

Repeat photographs were requested when the submitted images were insufficient for remote assessment. An unscheduled in-office examination was arranged for 6 of the 50 participants in the educational telemonitoring cohort (12.0%) because of suspected appliance instability, soft tissue concerns, uncertain screw position, inconclusive photographic findings or inadequate image quality requiring chairside verification.

These findings indicate that the photographic submission protocol was feasible among the selected families included in the educational telemonitoring cohort. However, photographic adequacy represented the ability to perform a predefined remote visual assessment and did not imply equivalence to a complete chairside clinical examination.

### 3.3 Recorded clinical complications

The primary outcome was determined using prospectively collected follow-up documentation, including scheduled and unscheduled clinical assessments, caregiver communications, remote photographic review, clinical records and the standardized final in-office examination performed in all participants. At least one recorded biological or technical complication was documented in 12 of 50 participants (24.0%) in the educational telemonitoring cohort and in 23 of 50 participants (46.0%) in the conventional cohort (unadjusted RR, 0.52; 95% CI, 0.29–0.93; *p* = 0.021). Recorded biological complications occurred in 8 of 50 participants (16.0%) in the educational telemonitoring cohort and 18 of 50 participants (36.0%) in the conventional cohort (unadjusted RR, 0.44; 95% CI, 0.21–0.93; *p* = 0.023). The most frequently documented biological findings were gingival inflammation, clinically relevant plaque accumulation around the appliance and minor mucosal irritation.

Recorded technical complications occurred in 6 of 50 participants (12.0%) in the educational telemonitoring cohort and 15 of 50 participants (30.0%) in the conventional cohort (unadjusted RR, 0.40; 95% CI, 0.17–0.95; *p* = 0.027). Activation-related problems were documented in 5 of 50 (10.0%) in the educational telemonitoring cohort and 13 of 50 participants (26.0%) in the conventional cohort (unadjusted RR, 0.38; 95% CI, 0.148–0.999; *p* = 0.037). The most frequently recorded technical findings were incorrect or uncertain screw activation and expander loosening.

Activation-related problems were included within the technical-complication category. Individual participants could experience both biological and technical complications. Therefore, the sum of the participants included in the separate complication categories exceeded the number of participants experiencing the composite primary outcome. Participants with multiple complications were counted only once in the composite analysis.

All effect estimates were unadjusted. Moreover, because the frequency and modality of interim assessment differed between the two follow-up pathways, minor or transient findings may have had different probabilities of detection and documentation. The observed differences therefore represent exploratory associations in the frequency of recorded complications and should not be interpreted as definitive evidence of a causal reduction in the true incidence of complications. The results are summarized in Table 2.

**Table 2:** Recorded clinical outcomes during active rapid maxillary expansion.

Outcome	Conventional cohort (*n* = 50)	Educational telemonitoring cohort (*n* = 50)	Unadjusted RR (95% CI)	*p*-value (Pearson's chi-square test)
Participants experiencing at least one recorded biological or technical complication, *n* (%)	23 (46.0)	12 (24.0)	0.52 (0.29–0.93)	0.021
Biological complications, *n* (%)	18 (36.0)	8 (16.0)	0.44 (0.21–0.93)	0.023
Technical complications, *n* (%)	15 (30.0)	6 (12.0)	0.40 (0.17–0.95)	0.027
Activation-related problems, *n* (%)	13 (26.0)	5 (10.0)	0.38 (0.148–0.999)	0.037

Risk ratios were calculated for the educational telemonitoring cohort relative to the conventional follow-up cohort. Participants could experience both biological and technical complications; therefore, the sum of the individual categories may exceed the number of participants experiencing the composite primary outcome. Activation-related problems were included within the technical-complication category. All estimates were unadjusted and should be interpreted as exploratory because follow-up pathway selection was non-randomized and interim outcome-assessment modalities differed between cohorts.

### 3.4 Activation adherence and treatment acceptance

Activation adherence was calculated using the daily caregiver activation logs and was verified against the clinically observed progression of the expansion screw during scheduled, unscheduled or final in-office assessments. Mean activation adherence was 94.8 ± 6.1% in the educational telemonitoring cohort and 89.3 ± 9.7% in the conventional follow-up cohort. The unadjusted mean difference, calculated as the educational telemonitoring cohort minus the conventional cohort, was 5.50 percentage points (95% CI, 2.32–8.68; *p* = 0.001).

The most frequently documented activation-related problems were missed activations, uncertainty regarding screw progression and incorrectly performed activations. In the educational telemonitoring cohort, weekly upper occlusal photographs provided an additional opportunity to identify uncertain screw progression and reinforce the caregiver's activation technique. However, because serial photographic verification was available only in the telemonitoring cohort, differential assessment of activation adherence between the two pathways could not be completely excluded.

At completion of active expansion, mean child-reported treatment acceptance was 4.2 ± 0.7 in the educational telemonitoring cohort and 3.7 ± 0.8 in the conventional cohort. The unadjusted mean difference was 0.50 points (95% CI, 0.21–0.79; *p* = 0.001).

Mean caregiver-reported treatment acceptance was 4.3 ± 0.6 in the educational telemonitoring cohort and 3.7 ± 0.8 in the conventional cohort, corresponding to an unadjusted mean difference of 0.60 points (95% CI, 0.32–0.88; *p* < 0.001). Mean caregiver confidence in appliance management was 4.3 ± 0.6 and 3.5 ± 0.9, respectively, with an unadjusted mean difference of 0.80 points (95% CI, 0.50–1.10; *p* < 0.001). Mean overall treatment satisfaction was 4.3 ± 0.6 in the educational telemonitoring cohort and 3.8 ± 0.7 in the conventional cohort, corresponding to an unadjusted mean difference of 0.50 points (95% CI, 0.24–0.76; *p* < 0.001).

Child- and caregiver-reported outcomes were assessed using study-specific, non-validated single-item 5-point Likert measures. These outcomes were therefore considered exploratory and may have been influenced by expectations regarding the selected follow-up pathway, social-desirability bias and the absence of participant blinding. Furthermore, no adjustment for multiple comparisons was applied.

Accordingly, the *p*-values for these secondary outcomes should be interpreted cautiously. The results are summarized in Table 3.

**Table 3:** Activation adherence and treatment acceptance outcomes.

Outcome	Conventional follow-up cohort (*n* = 50)	Educational telemonitoring cohort (*n* = 50)	Unadjusted mean difference	95% CI	*p*-value
Activation adherence, %	89.3 ± 9.7	94.8 ± 6.1	5.50	2.32–8.68	0.001
Child-reported treatment acceptance	3.7 ± 0.8	4.2 ± 0.7	0.50	0.21–0.79	0.001
Caregiver-reported treatment acceptance	3.7 ± 0.8	4.3 ± 0.6	0.60	0.32–0.88	<0.001
Caregiver confidence in appliance management	3.5 ± 0.9	4.3 ± 0.6	0.80	0.50–1.10	<0.001
Overall treatment satisfaction	3.8 ± 0.7	4.3 ± 0.6	0.50	0.24–0.76	<0.001

Data are presented as mean ± standard deviation. Mean differences were calculated as the educational telemonitoring cohort minus the conventional follow-up cohort. Activation adherence was calculated from caregiver activation logs and verified against the clinically observed progression of the expansion screw. Treatment acceptance, caregiver confidence and overall satisfaction were assessed using study-specific, non-validated single-item 5-point Likert scales, with higher scores indicating greater acceptance, confidence or satisfaction. All estimates were unadjusted. No adjustment for multiple comparisons was applied; therefore, these secondary outcomes should be interpreted as exploratory.

### 3.5 In-Office follow-Up and unscheduled visits

The scheduled follow-up pathway differed between the two cohorts. Participants in the conventional cohort attended weekly scheduled in-office follow-up visits during active expansion. Participants in the educational telemonitoring cohort underwent weekly remote photographic review and a scheduled in-office examination at completion of active expansion. When active expansion continued beyond four weeks, an additional in-office reassessment was scheduled at approximately four weeks. In both cohorts, earlier unscheduled in-office examinations were arranged whenever clinically indicated.

At least one unscheduled in-office visit was recorded for 10 of 50 participants (20.0%) in the conventional follow-up cohort and for 6 of 50 participants (12.0%) in the educational telemonitoring cohort. The unadjusted risk ratio for the educational telemonitoring cohort relative to the conventional cohort was 0.60 (95% CI, 0.24–1.53; *p* = 0.275). Therefore, no statistically significant between-cohort difference in the proportion of participants requiring at least one unscheduled in-office visit was observed.

In the conventional follow-up cohort, the main reasons for unscheduled assessment were caregiver-reported appliance loosening, uncertainty regarding screw activation and soft tissue irritation requiring chairside verification. In the educational telemonitoring cohort, unscheduled visits were mainly prompted by inconclusive photographic findings, suspected appliance instability, soft tissue concerns, uncertain screw position or inadequate image quality requiring clinical verification.

Because the indications for unscheduled assessment differed partly according to the selected follow-up pathway, the frequency of unscheduled visits should not be interpreted as a direct measure of complication frequency. In particular, an unscheduled visit in the telemonitoring cohort could be arranged because remote assessment was inconclusive or image quality was inadequate, even when no complication was subsequently confirmed.

Two participants in the conventional follow-up cohort experienced a temporary interruption of the prescribed activation protocol, compared with no participants in the educational telemonitoring cohort (Fisher's exact test, *p* = 0.495). No participant discontinued active expansion or was lost to follow-up in either cohort.

No delayed detection of a clinically relevant biological or technical complication was documented in either cohort. However, because the sample size was limited and no delayed events occurred, this finding should not be interpreted as definitive evidence of equivalence or comparative safety between the two follow-up pathways.

The actual number of scheduled and total in-office visits per participant was not prospectively quantified or analyzed. Consequently, no formal comparison of overall visit burden, chairside time, clinical resource utilization, caregiver travel or time away from school or work was performed. The characteristics of the follow-up pathways and the recorded unscheduled visits are summarized in Table 4.

**Table 4:** Follow-up pathways, unscheduled in-office visits and treatment interruptions during active rapid maxillary expansion.

Outcome	Conventional cohort (*n* = 50)	Educational telemonitoring cohort (*n* = 50)	Effect estimate	*p*-value
Scheduled follow-up pathway	Weekly scheduled in-office examinations throughout the active expansion phase	Weekly remote photographic review and a scheduled in-office examination at completion of active expansion; an additional in-office reassessment at approximately 4 weeks when expansion remained ongoing	Not applicable	
Participants requiring at least one unscheduled in-office visit, *n* (%)	10 (20.0)	6 (12.0)	RR 0.60 (95% CI, 0.24–1.53)	0.275
Main reason for unscheduled visits	Appliance loosening, activation concerns or soft tissue irritation requiring chairside verification	Unclear photographic findings, suspected appliance instability, soft tissue irritation, uncertain screw position or inadequate image quality	Not applicable	—
Temporary treatment interruption, *n* (%)	2 (4.0)	0 (0.0)	Not estimable	0.495
Treatment discontinuation, *n* (%)	0 (0.0)	0 (0.0)	Not estimable	
Delayed detection of clinically relevant complications, *n* (%)	0 (0.0)	0 (0.0)	Not estimable	—

Risk ratios were calculated for the educational telemonitoring cohort relative to the conventional follow-up cohort. An unscheduled visit was defined as an additional chairside assessment prompted by caregiver concern, suspected biological or technical complications, inconclusive remote findings or inadequate image quality. Because the indications for unscheduled assessment differed between pathways, this outcome should not be interpreted as a direct measure of complication frequency. Fisher's exact test was used for temporary treatment interruption. All estimates were unadjusted and exploratory.

### 3.6 Summary of results

The educational telemonitoring pathway was feasible among the selected participating families. Caregivers submitted 94.0% of the expected photographic sets and 92.6% of the submitted sets met the predefined criteria for remote visual assessment.

In unadjusted between-cohort comparisons, the educational telemonitoring cohort showed a lower frequency of recorded composite, biological, technical and activation-related complications than the conventional follow-up cohort. Mean activation adherence and child- and caregiver-reported treatment-acceptance scores were also higher in the educational telemonitoring cohort.

The proportion of participants requiring at least one unscheduled in-office visit did not differ significantly between the two cohorts. No treatment discontinuation, loss to follow-up or delayed detection of a clinically relevant complication was documented in either cohort.

These findings should be interpreted cautiously. Follow-up pathway selection was based on caregiver preference, comparative effect estimates were unadjusted and the frequency and modality of interim assessment differed between cohorts. Therefore, the observed differences represent exploratory associations in prospectively recorded outcomes and may reflect both the characteristics of the follow-up pathways and measured or unmeasured differences between participating families. They should not be interpreted as evidence that the educational telemonitoring pathway caused a reduction in the true incidence of complications.

## 4 Discussion

This prospective, preference-based comparative cohort study evaluated two follow-up pathways during the active phase of rapid maxillary expansion in children (69–83). The combined educational and telemonitoring pathway was feasible among the selected participating families, as shown by the high photographic submission and adequacy rates. In unadjusted comparisons, the educational telemonitoring cohort had fewer recorded biological and technical complications, higher activation adherence and higher child- and caregiver-reported treatment-acceptance scores than the conventional weekly in-office follow-up cohort (84–95). The proportion of participants requiring at least one unscheduled in-office visit did not differ significantly between cohorts, and no delayed detection of a clinically relevant complication was documented.

These findings should not be interpreted as evidence that telemonitoring caused a reduction in the true incidence of complications. Follow-up pathway selection was based on caregiver preference, the comparative analyses were unadjusted and the frequency and modality of interim assessment differed between cohorts. Participants in the conventional cohort underwent weekly chairside examinations, whereas participants in the educational telemonitoring cohort were primarily evaluated through weekly intraoral photographs and less frequent in-office assessments. Consequently, minor or transient findings may have had different probabilities of detection and documentation. The standardized final in-office examination performed in all participants improved the comparability of the final assessment but could not identify events that had resolved before treatment completion (96–106).

Caregiver participation is particularly important during rapid maxillary expansion because screw activation, oral-hygiene supervision and recognition of appliance-related problems frequently occur outside the clinical setting (107–115). The educational telemonitoring pathway combined several components, including verbal and illustrated instructions, a photographic mouth opener, standardized weekly home photography, clinician-led image review and individualized remote feedback. These components may have reinforced caregiver attention to screw progression, oral hygiene and visible appliance or soft tissue changes. However, because the educational and telemonitoring elements were delivered as a combined package, the present study cannot determine which component or combination of components contributed to the observed associations (116, 117).

The higher activation adherence observed in the educational telemonitoring cohort may be related to repeated caregiver engagement and the opportunity to clarify activation-related uncertainty between in-office examinations. Nevertheless, serial upper occlusal photographs were available only in the telemonitoring cohort and provided an additional source for evaluating screw progression. Differential verification of adherence therefore cannot be excluded, even though the standardized adherence calculation was based on caregiver activation logs and clinically observed screw progression in both cohorts (118–124).

Higher child- and caregiver-reported acceptance, caregiver confidence and overall treatment satisfaction were also observed in the educational telemonitoring cohort. Structured information and continued contact with the orthodontic team may have reduced caregiver uncertainty and increased the perceived level of support during treatment. However, these outcomes were measured using study-specific, non-validated single-item measures. In addition, participants and caregivers were aware of the selected pathway and families who actively chose telemonitoring may have had more favorable expectations regarding this form of follow-up. These patient- and caregiver-reported findings should therefore be considered exploratory (125–135).

The results support the use of telemonitoring as an adjunct to clinical assessment rather than as a complete replacement for chairside examination. Standardized intraoral photographs may permit visual assessment of screw visibility, activation progression, oral-hygiene conditions, palatal and gingival tissues and visible changes in appliance position. However, remote images cannot reproduce palpation, direct mobility testing, assessment of cement integrity or a complete intraoral examination.

In the present pathway, remote review therefore functioned primarily as a monitoring and triage strategy, with in-office reassessment arranged whenever images were inadequate, findings were inconclusive or a biological or technical complication was suspected (136–149).

The proposed pathway may have practical relevance in clinical settings where dedicated commercial remote-monitoring systems are unavailable. It was based on commonly available smartphones, standardized educational material, a simple photographic mouth opener, secure image transmission and clinician-led review (150–161). However, the present study did not quantify the actual number of scheduled and total in-office visits per participant, clinician review time, caregiver workload, travel requirements, school or work absence or treatment-related costs. Therefore, the pathway should be described as pragmatic and non-commercial, but not as proven to reduce clinical resource use, visit burden or costs (70, 162–164).

The preference-based design reflects how families may choose between different follow-up options in routine clinical practice and provides information regarding real-world feasibility and acceptability. At the same time, caregiver preference may have introduced systematic differences between cohorts. Families selecting telemonitoring may have had greater motivation, digital competence, health literacy, availability or willingness to participate actively in treatment, whereas families selecting conventional follow-up may have preferred direct professional supervision. These unmeasured characteristics could have influenced adherence, oral-hygiene behavior, reporting of symptoms and questionnaire responses independently of the selected pathway (165–178).

The main strengths of the study were its prospective data collection, complete primary-outcome follow-up, predefined complication categories, standardized final in-office examination for all participants and evaluation of both clinical and patient-centered outcomes. The inclusion of a detailed home-photography protocol and predefined criteria for repeat photographs and chairside reassessment also improves the reproducibility of the telemonitoring pathway. Nevertheless, the observational design provides a lower level of causal evidence than randomized allocation and the findings should be interpreted within the methodological limitations described below (179–184).

### 4.1 Limitations

This study has several limitations. First, cohort allocation was not randomized but was determined by caregiver preference. Families selecting the educational telemonitoring pathway may have differed from those selecting conventional weekly in-office follow-up in motivation, digital competence, health literacy, socioeconomic circumstances, availability, expectations or willingness to participate actively in treatment. These characteristics were not comprehensively measured and may have influenced activation adherence, oral-hygiene behavior, symptom reporting, complication documentation and questionnaire responses independently of the selected follow-up pathway. Therefore, selection bias and residual confounding cannot be excluded.

Quota-based consecutive recruitment was conducted separately within each caregiver-selected pathway to obtain equal cohort sizes. Although participants were consecutively enrolled within each pathway, recruitment was closed after the prespecified quota of 50 participants had been reached. Families selecting a pathway after the corresponding quota had been reached were not enrolled. Consequently, the study population represented two pathway-specific consecutive samples rather than a single consecutive clinical sample and additional selection effects may have been introduced.

Second, no formal *a priori* sample size calculation was performed and the study size was based on a pragmatic prespecified recruitment target. Only 35 participants experienced the composite primary outcome, limiting the number of covariates that could be incorporated into a multivariable model without increasing the risk of model instability and overfitting. Moreover, as noted above, several caregiver-level factors potentially associated with both pathway selection and patient- or caregiver-reported outcomes were not comprehensively measured; therefore, adjustment using only the available demographic and clinical variables would not have eliminated the major risk of residual confounding. Accordingly, all comparative effect estimates were unadjusted and exploratory and residual confounding may account for at least part of the observed between-cohort differences.

The sample-size sensitivity analysis further indicated that the achieved cohort sizes were primarily capable of detecting relatively large between-cohort differences; smaller effects and uncommon outcomes may therefore have remained undetected.

Third, the frequency and modality of interim assessment differed between the two cohorts. Participants in the conventional follow-up cohort underwent weekly chairside examinations, whereas participants in the educational telemonitoring cohort primarily underwent weekly photographic review and less frequent in-office assessment. Minor or transient biological and technical findings may therefore have had different probabilities of detection and documentation. Although all participants underwent the same standardized final in-office clinical examination, this assessment could not identify events that had resolved before completion of active expansion. Consequently, the lower frequency of recorded complications in the telemonitoring cohort may reflect both a true difference between the pathways and differential outcome ascertainment. Although complications were documented using the clinical information available within each pathway, the database did not prospectively assign each event to a single mutually exclusive detection source. Therefore, a valid stratified sensitivity analysis restricted to complications detected at the common final examination could not be performed. This prevents quantification of the extent to which the observed between-cohort difference may have resulted from differential ascertainment. Activation adherence may also have been affected by differential assessment. Adherence was calculated from caregiver activation logs and verified against clinically observed screw progression in both cohorts. However, serial upper occlusal photographs provided an additional source of information regarding screw progression only in the educational telemonitoring cohort. This difference may have influenced the identification and correction of activation-related problems.

Fourth, participants, caregivers and treating clinicians could not be blinded to the selected follow-up pathway. Complete masking of the examiners reviewing the final clinical findings could not be guaranteed because clinical records and photographic documentation differed between cohorts. No formal inter-examiner reliability analysis was performed. Although disagreements were resolved through discussion and consensus, performance, detection and reporting bias cannot be excluded.

Fifth, child- and caregiver-reported outcomes were assessed using study-specific, non-validated single-item 5-point Likert measures. These measures had not undergone formal psychometric validation or test–retest reliability assessment. Because each construct was assessed using a single item, internal consistency could not be evaluated using coefficients such as Cronbach's alpha. These outcomes may also have been influenced by expectation, recall, social-desirability, and reporting biases. In addition, no adjustment for multiple comparisons was applied to the secondary outcomes, increasing the possibility of chance findings. These results should therefore be considered exploratory.

Sixth, the study was conducted at a single center and included children aged 7–11 years in mixed dentition whose families were able to comply with their independently selected follow-up pathway. Availability of a suitable smartphone and willingness to acquire and securely submit intraoral photographs were required only for families selecting educational telemonitoring. These requirements may have introduced digital-selection bias and limit the generalizability of the findings to families with limited access to digital technologies, lower digital competence or different socioeconomic and healthcare settings.

Seventh, the educational and telemonitoring components were delivered as a combined package. The study cannot determine whether the observed associations were related to the illustrated educational material, the photographic mouth opener, weekly image submission, clinician feedback, increased caregiver attention or the combined effect of these components.

Finally, the actual number of scheduled and total in-office visits per participant was not analyzed. Therefore, the study does not demonstrate a reduction in overall visit burden or clinical resource utilization. No formal assessment of costs, clinician review time, caregiver workload, travel, school or work absence or cost-effectiveness was performed. Follow-up was limited to the active expansion phase and long-term skeletal and dentoalveolar stability, appliance retention, subsequent treatment outcomes and delayed complications were not evaluated.

### 4.2 Future perspectives

Future studies should use multicenter designs and include families with broader socioeconomic backgrounds, different levels of digital access and varying degrees of digital competence to improve the generalizability of the findings. Caregiver motivation, health literacy, expectations, technological confidence and practical barriers to remote participation should be measured prospectively and considered as potential confounding factors.

Randomized controlled trials would be required to estimate the causal effectiveness of educational telemonitoring compared with conventional in-office follow-up. However, preference-based observational studies may remain useful for evaluating acceptability, feasibility and implementation under routine clinical conditions. Future comparative studies should therefore consider both randomized and preference-sensitive designs, depending on the primary research objective.

To reduce differential outcome ascertainment, future studies should apply identical schedules and standardized methods for outcome evaluation in both groups. This could include common in-office examinations at predefined time points, independent and blinded outcome assessment whenever feasible, validated clinical criteria for biological and technical complications and documentation of the timing and source of each detected event. Clinically relevant complications requiring chairside intervention, appliance repair, treatment interruption or modification of the activation protocol should also be analyzed separately from minor or transient findings.

Future research should use validated pediatric and caregiver-reported outcome measures and should prespecify primary and secondary outcomes, sample-size calculations, statistical adjustment for relevant confounders and strategies for multiple comparisons. Larger samples would permit adjusted analyses and more precise evaluation of uncommon adverse events and safety-related outcomes.

Additional studies should distinguish the effects of the individual components included in the combined pathway, such as illustrated educational material, photographic aids, weekly image submission and clinician feedback. Factorial or multi-arm designs may help determine whether the observed benefits are attributable primarily to education, remote monitoring, repeated caregiver engagement or their combined effect.

Future implementation studies should also quantify the actual number of scheduled and unscheduled in-office visits, clinician review time, caregiver workload, travel requirements, school and work absence, data-security requirements and direct and indirect costs. Formal health-economic analyses are needed before the pathway can be considered resource-saving or cost-effective.

Finally, longer follow-up is required to evaluate retention, skeletal and dentoalveolar stability, subsequent orthodontic treatment, delayed complications and the sustainability of caregiver adherence. Comparative research should also assess simple clinician-reviewed photographic protocols against commercial and artificial intelligence-supported remote-monitoring systems while maintaining appropriate clinical oversight and predefined criteria for chairside reassessment.

## 5 Conclusions

In this single-center, prospective, preference-based comparative cohort study, the combined educational and weekly telemonitoring pathway was feasible among selected families during the active phase of rapid maxillary expansion. High photographic submission and adequacy rates indicated that standardized caregiver-taken intraoral photographs could support clinician-led remote visual assessment and triage.

In unadjusted comparisons, the educational telemonitoring cohort showed fewer recorded biological and technical complications, higher activation adherence and greater child- and caregiver-reported treatment acceptance than the conventional weekly in-office follow-up cohort. The proportion of participants requiring at least one unscheduled in-office visit did not differ significantly between the two pathways and no delayed detection of a clinically relevant complication was documented.

These findings should be interpreted as exploratory. Caregivers independently selected the follow-up pathway, no adjusted comparative analysis was performed and the frequency and modality of interim assessment differed between cohorts. Consequently, the observed differences may have been influenced by selection bias, residual confounding, differential outcome ascertainment and differences in caregiver engagement. The results should not be interpreted as evidence that the educational telemonitoring pathway caused a reduction in the true incidence of complications or as proof of equivalence in safety.

Telemonitoring should therefore be considered an adjunct to, rather than a replacement for, clinical examination. Its implementation should include structured caregiver education, standardized image acquisition, secure data transmission, timely clinician review, appropriate family selection and predefined criteria for in-office reassessment. Randomized and multicenter studies using identical outcome-assessment schedules are required to confirm clinical effectiveness, safety, generalizability and resource implications.

## Data Availability

The raw data supporting the conclusions of this article will be made available by the authors, without undue reservation.
