# Supplementary material for: Educational support and weekly telemonitoring during rapid maxillary expansion in children: a prospective preference-based comparative cohort study
Source: Front Dent Med. 2026 Sep 28;7:1959672. doi: 10.3389/fdmed.2026.1959672 (PMC13654767; doi:10.3389/fdmed.2026.1959672)
Supplement: Supplementary file 1 [file Datasheet1.pdf]

Supplementary Material

1 Supplementary Data

Appendix A: *Home Intraoral Photography Protocol for Rapid Maxillary Expansion Telemonitoring*

A1. Purpose

This protocol standardizes home intraoral photographs in pediatric patients undergoing rapid maxillary expansion to support remote evaluation of appliance status, oral hygiene, soft tissue conditions, and activation progression.

A2. Equipment Required

- a smartphone camera;
- the provided photographic mouth opener;
- adequate lighting, preferably natural daylight or strong indoor light. Flash may be used if ambient lighting is insufficient.

A3. General Instructions

Before taking photographs:

- the child should brush teeth thoroughly;
- the caregiver should wash hands;
- the mouth opener should be cleaned;
- the child should sit upright and face the camera;
- photographs should be taken once per week, preferably on the same day;
- the camera should be positioned approximately 20–30 cm from the mouth;
- photographs should be taken in horizontal orientation;
- the expander should be clearly visible.

A4. Mandatory Photographic Set

Each weekly photographic set includes four images.

1. Frontal intraoral view: the child bites gently in light occlusion. This image is used to assess the incisors, midline, general hygiene and visible inflammation.
2. Upper occlusal view: the mouth opener is placed and the camera is positioned above the mouth. This image must show the expander, screw, palatal tissues and maxillary arch. It is used to evaluate activation progression, plaque accumulation and appliance stability.
3. Right lateral view: the mouth opener is positioned to expose the right posterior segment. This image is used to assess bands, soft tissue irritation, appliance stability and hygiene.
4. Left lateral view: the same procedure is repeated on the left side to allow bilateral comparison.

A5. Image Quality Criteria

A photographic set is considered adequate when:

1. The images are not blurred;
2. the expander is centered;
3. the screw is visible;

4. the palatal tissues are visible;
5. no shadows obscure the appliance;
6. fingers or lips do not cover the area of interest.

When one or more images are unclear, the caregiver is asked to repeat the relevant photograph.

A6. File Naming and Submission

Photographs should be named using the pseudonymized format Patient ID_Date_View (for example: P034_2026-02-01_UpperOcclusal.jpg) and submitted through the approved

secure clinical platform or encrypted institutional email in accordance with privacy procedures.

A7. Red Flag Checklist for Caregivers

Caregivers should contact the orthodontic team when any of the following signs are present:

1. bleeding;
2. ulceration;
3. swelling;
4. marked redness;
5. persistent pain;
6. broken or detached appliance components;
7. inability to activate the screw;
8. uncertainty regarding screw activation;
9. a sudden change in appliance position;
10. image quality that prevents adequate visualization.

A8. Clinical Evaluation Grid for Orthodontist (Table A1)

A9. Potential Practical Advantages

When appropriately implemented, this protocol may support caregiver understanding, structured clinical documentation, timely triage, and reinforcement of activation and oral-hygiene instructions. These potential advantages require confirmation through appropriately designed comparative and economic studies.

2 Supplementary Figures and Tables

2.1 Supplementary Figures

Table A1. Standardized clinical evaluation grid for weekly photographic review.

Assessment domain	Classification	Possible action
Oral hygiene	Good<br>Moderate<br>Poor	No action<br>Reinforce hygiene instructions<br>Schedule in-office visit
Soft tissues	Normal<br>Inflamed<br>Ulcerated	No action<br>Remote advice<br>Schedule in-office visit
Screw visibility	Clearly visible<br>Partially visible<br>Not visible	No action<br>Request new occlusal photograph<br>Schedule in-office visit
Screw activation progression	Adequate<br>Doubtful<br>Inadequate	No action<br>Reinforce activation instructions<br>Schedule in-office verification
Appliance stability	Stable<br>Doubtful<br>Suspected loosening	No action<br>Request additional photographs<br>Schedule unscheduled in-office visit
Reported pain or discomfort	Absent<br>Mild<br>Moderate or severe	No action<br>Remote advice<br>Schedule in-office visit
Image quality	Adequate<br>Partially adequate<br>Inadequate	No action<br>Repeat photographs<br>Schedule in-office visit
Red flags	Absent<br>Present	No action<br>Immediate caregiver contact and possible unscheduled in-office visit
Final decision	No action required<br>Remote instruction<br>Repeat photographs<br>Unscheduled in-office visit	Record the decision in the participant file

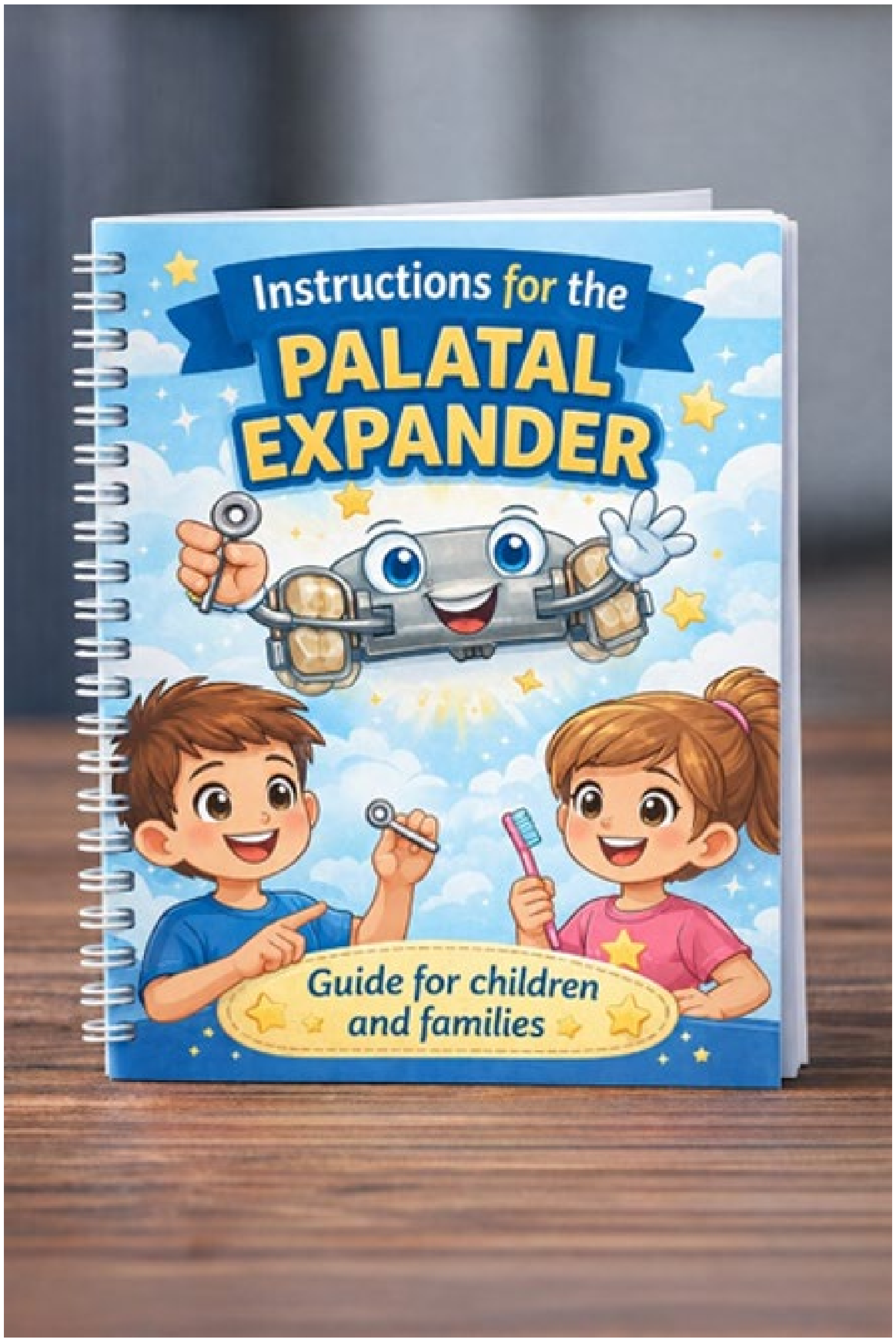

Supplementary Figure A1. Intraoral photography instructions provided to caregivers to standardize image acquisition during weekly telemonitoring.

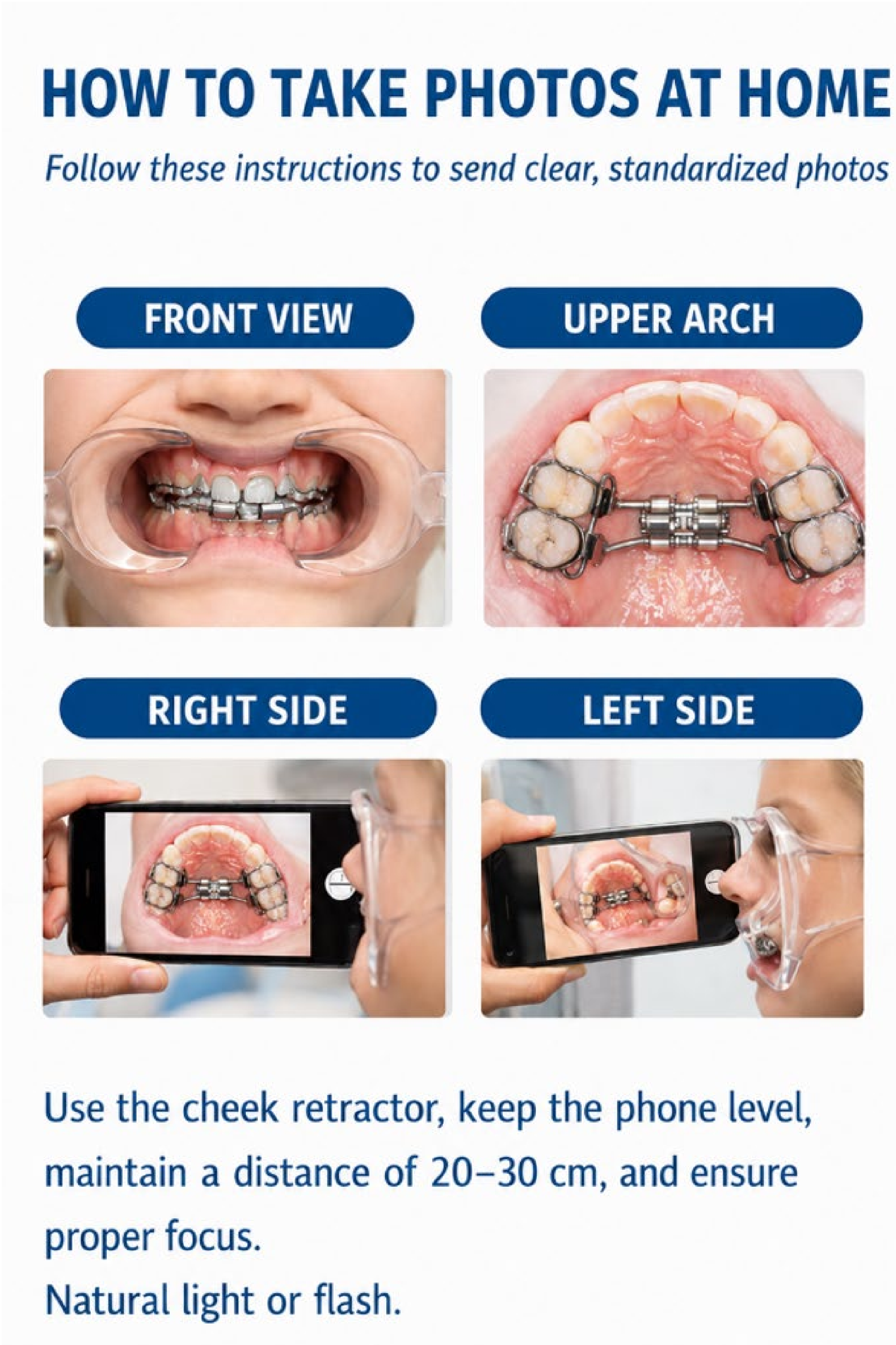

Supplementary Figure A2. Intraoral photography instructions provided to caregivers to standardize image acquisition during weekly telemonitoring.
